# Patients’ perspective on emergency treatment of ophthalmologic diseases during the first phase of SARS-CoV2 pandemic in a tertiary referral center in Germany – the COVID-DETOUR questionnaire study

**DOI:** 10.1186/s12886-021-02054-7

**Published:** 2021-08-16

**Authors:** Christoph Ehlken, Constantin von Medem, Maya Lüdemann, Anna Maria Kirsch, Johann Baptist Roider

**Affiliations:** 1 Klinik für Ophthalmologie des Universitätsklinikums Schleswig Holsteins, Campus Kiel, Kiel, Germany; 2 Augenklinik Klinikum Kassel, Kassel, Germany

**Keywords:** COVID, Emergency, Pandemic, Delay in treatment

## Abstract

**Background:**

During the first wave of the COVID-19 pandemic, the need of treatment of urgent ophthalmological diseases and the possible risk of a SARS-CoV-2 infection had to be weighed against each other. In this questionnaire study, we aimed to analyze potential barriers and patients’ health beliefs during and after the lockdown early 2020 in a tertiary referral center in Kiel, Germany.

**Methods:**

Patients admitted for the treatment of urgent ophthalmic diseases between March 1st, 2020, and June 3rd, 2020, were asked to participate in a questionnaire study. After informed consent was obtained, patients were interviewed using a standardized questionnaire which addressed aspects of their medical history, their health beliefs concerning the COVID-19 pandemic and barriers on their way to the treatment center. The study group was subdivided into two subgroups, depending on the occurrence of their symptoms, before and after the lockdown was ended on April 20th, 2020.

**Results:**

Ninety-three patients were included, 43 in subgroup A (before April 20th) and 50 in subgroup B (April 20th or later). Retinal disorders were the most common causes for admission (approximately 60%).. Only 8 patients (8.6%) experienced a delay between their decision to visit a doctor until the actual examination. Every fourth patient was afraid of a COVID-19 infection, and expected a higher likelihood for an infection at the hospital. Patients with comorbidities tended to be more likely to be afraid of an infection (correlation coefficient 0.183, *p* = 0.0785) and were significantly more likely to be concerned about problems with organizing follow-up care (corr. Coefficient 0.222, *p* = 0.0328). Higher age was negatively correlated with fear of infection (corr. Coefficient − 0.218, *p*-value 0.034).

**Conclusion:**

In this questionnaire study, only a minority of patients indicated a delay in treatment, regardless of whether symptoms occurred before or after the lockdown before April 20th, 2020. While patients with comorbidities were more concerned about infection and problems during follow-up care, patients of higher age – who have a higher mortality – were less afraid. Protection of high-risk groups should be prioritized during the SARS-CoV-2 pandemic.

**Trial registration:**

The study was registered as DRKS00021630 at the DRKS (Deutsches Register Klinischer Studien) before the conduction of the study on May 5th, 2020.

**Supplementary Information:**

The online version contains supplementary material available at 10.1186/s12886-021-02054-7.

## Key messages

- Several studies reported the underutilization of emergency units during the first phase of the SARS-CoV-2 pandemic in 2020, resulting delayed diagnosis and treatment with more progressed stages of disease, e.g. in patients with retinal detachment.
- In this questionnaire study, only a minority of patients indicated delays from symptoms to ophthalmological examination or treatment in our tertiary referral center in Germany.
- More than every fourth patient was concerned of a COVID-19 infection. While patients with comorbidities were more concerned, older patients – though being at a higher risk for severe course of a COVID disease – were less afraid of an infection.

## Background

In early 2020, the SARS-CoV-2 (severe acute respiratory syndrome, coronavirus 2) spread quickly across the globe. The virus was first described in a local outbreak in Wuhan, China, in December 2019. However, it was soon detected in multiple countries and declared a pandemic by the WHO on March 11th, 2020. As of mid-December 2020, more than 70 million infections and 1.6 million deaths related to COVID-19, the disease caused by SARS-CoV-2, were counted in 191 countries. It caused considerable uncertainty among all parts of society. Governments restricted liberties, health care providers were burdened with the task to prioritize access to the health system, and patients had to decide if their symptoms required urgent diagnosis or therapy.

In Europe, Italy was heavily affected in early 2020, with a health care system on the brink of decompensation. Intensive care units operated at full capacity only 6 weeks after the first confirmed case [1, 2]. Reports by Italian scientists helped other countries estimate risks and establish protocols for dealing with the expected increase of COVID patients. Multiple countries went into lockdown, though exact procedures differed between and even within countries.

Similar to other medical areas, ophthalmologic societies published recommendations for prioritizing examinations and surgery, such as the American Academy of Ophthalmology (https://www.aao.org/headline/list-of-urgent-emergent-ophthalmic-procedures), or the Royal College of Ophthalmologists in the UK (http://rcophth.ac.uk/about/rcophth-guidance-on-restoring-ophthalmology-services/). Updated guidelines were suggested in order to prevent unnecessary visits on the one hand, but ensure adequate therapy on the other [3]. Ophthalmologists (as well as dentists and otolaryngologists) are considered to have a higher risk of infection compared to other disciplines due to the proximity of patient and doctor during the examination. In a survey among German ophthalmologists, 80% considered themselves to be at a high risk for a COVID-19 infection [4].

In Germany, the first case of COVID-19 was confirmed on January 24th, 2020. Southern federal states were affected earlier and more severely compared to the northern ones. Federal guidelines differed between states concerning the intensity of a general lockdown and the feasibility of elective surgery. A questionnaire study of 1190 health care professionals in Germany reported, that during the first wave of COVID-19 (approximately mid-March to mid-April) patient numbers were reduced to approximately 30% of pre-pandemic levels, and hospital beds were reserved for emergency patients in 70% of the participating centers [5]. However, less patients were admitted with ophthalmic emergencies, such as rhegmatogenous retinal detachment (RRD), perforating trauma, central retinal arterial occlusion or acute ischemic optic neuropathy. The respondents stated that those lower numbers were caused by institutional guidelines (e.g. only emergencies accepted), but also by patients cancelling their appointments.

In Germany, patients with acute ophthalmologic diseases generally approach a referral ophthalmologist, who then decides whether further surgical treatment and/or hospitalization at a tertiary center is necessary. In that case, the patient is referred to the tertiary center (such as the study center in Kiel) for further treatment. During the first lockdown, patients were faced with restricted access to the health system. Private practices were partially closed, or only a limited number of patients was allowed due to distancing rules. Public transport was restricted. As people were told to avoid social contacts, support by relatives or friends could be limited in case a patient needed help to reach their physician.

In this study, we aimed to investigate the patients’ perspective of delays and barriers in diagnosis and/or treatment of ophthalmologic emergencies due to COVID-19 in a referral tertiary university hospital in northern Germany.

## Methods

### Patient eligibility and recruitment

Patients were eligible, if the following predefined criteria were met: patient signed informed consent form; patient presented with a symptomatic and acute ophthalmological disease; the disease required treatment as an inpatient; the patient was admitted to the ward between March 1st and June 3rd 2020.

During the prospective phase (May 7th to June 3rd), informed consent was confirmed during the stay at the hospital and the questionnaire was completed with the help of a member of the study group. Patients admitted between March 1st and May 6th (retrospective phase) were identified using an automatic search in the hospital’s electronic charts. Patients’ charts were manually reviewed regarding the diagnosis and emergency admissions were identified. Suitable patients were informed of the study by telephone or during planned control examinations. After informed consent was given, the questionnaire was completed with the help of a member of the study group. Telephone interviews were performed by the same interviewer (Josefin Kohn) between June 9th and September 30th, 2020.

### Questionnaire layout

The questionnaire addressed different areas: the diagnosis, time frames (onset of symptoms, time to a first contact with a referral ophthalmologist or general physician, time to hospital admission), patients’ attitude towards personal risk factors, barriers and fear of infection, and medical history. For questions answered with likert scales, a score of 3 or more (“in part”, “mostly agree” “totally agree”) was considered as indicative for a subjective burden. The questionnaire is provided as electronic supplementary information (see online resource 1).

### Dynamics of recommendations for elective and emergency treatment

In the study center, patients referred to the hospital with *urgent* medical conditions were accepted and examined as usual during the time of the study regardless of the general lockdown. Recommendations for treatment of *elective* patients changed during the course of the pandemic. In early March 2020, it was decided to delay any *elective* outpatients visits and surgery, and a general lockdown restricted patient mobility. After the lockdown was ended on April 20th, 2020, admission of elective patients was again allowed, following the university hospitals recommendations. To reflect this dynamic, the study group was subdivided into two subgroups (before April 20th, 2020, and after). Telemedicine was not available at the study center during the time of the study.

### Statistical analysis

Descriptive statistics were used to describe the study groups. Values are displayed as mean and standard deviation or number and percentages, respectively. For comparison between groups, t-test was used for continuous variables, Mann-Whitney-U test and Wilcoxon test were used where appropriate. Fisher’s exact test was used to compare discrete variables. Spearman correlation was used to analyze correlation of nonparametric variables.

Statistical analysis was performed using R 4.03 [6] and the EZR package [7]. *P* values of < 0.05 were considered statistically significant.

## Results

### Study group

One-hundred and seven patients signed informed consent. Of those, 59 were prospectively recruited and 48 were retrospectively included. Ten patients in the retrospective group were not available for the telephone interview and excluded from analysis. Four patients (2 in prospective group, 2 in retrospective group) were excluded, as the interview revealed that they were not symptomatic or treated as emergency patients. Consequently, 93 questionnaires (57 prospective, 36 retrospective) were included in the study. Demographics of the study group and subgroups are shown in Table 1.

**Table 1:** Study group demographics

	Total study group	During lockdown (before April 20th)	After lockdown (after April 20th)	*p*-value
group size	93	43	50	
age	65,3 +/− 13,9	65,6 +/− 13,8 [30;94]	65,0 +/− 14,1 [23;88]	0,8314
female	43 (46,2%)	23 (53,5%)	20 (40,0%)	0,216

In general, retinal disorders were the most common cause leading to hospital admission with more than 60% of cases, followed by corneal infections. Retinal detachment was the most prominent singular cause for emergency treatment. Diseases leading to admission are shown in Table 2.

**Table 2:** Diseases leading to hospital admission

Area	Diagnosis	ICD-10 code	Total study group no. (% of all)	Before April 20th	From April 20th
**Retinal disorders**	all		58 (62.4%)	26 (60.5%)	32 (64.0%)
	retinal detachment	H33.0, H33.3, H33.4	39 (41.9%)	19 (44.2%)	20 (40.0%)
	subretinal hemorrhage / AMD	H35.30	2 (2.2%)	1 (2.3%)	1 (2.0%)
	endophthalmitis	H44.1	4 (4.3%)	1 (2.3%)	3 (6.0%)
	vascular disorders: RVO / RAO / macroaneursym with subretinal hemorrhage	H34.0, H34.1, H34.2, H34.8, H35.6	12 (12.9%)	4 (9.3%)	8 (8.0%)
	macular hole	H35.38	1 (1.1%)	1 (2.3%)	0
**Corneal disorders**	all		13 (14.0%)	7 (16.3%)	6 (12.0%)
	keratitis, ulcus	H16.0, H16.2, H16.3, H19.1, B02.3, H19.2	12 (12.9%)	6 (14.0%)	6 (12.0%)
	corneal transplant failure	T86.83	1 (1.1%)	1 (2.3%)	0
**NO/orbit disorders**	all		10 (10.8%)	5 (11.6%)	5 (10.0%)
	neuritis	H46	3 (3.2%)	2 (4.7%)	1 (2.0%)
	AION	H47.0	6 (6.5%)	3 (7.0%)	3 (6.0%)
	infection	H04.3	1 (1.1%)	0	1 (2.0%)
**IOP disorders**	all		5 (5.4%)	1 (2.3%)	4 (8.0%)
	IOP decompensation	H40.5	4 (4.3%)	1 (2.3%)	3 (6.0%)
	bleb infection	H59.8	1 (1.1%)	0	1 (2.0%)
**others**	all		7 (7.5%)	4 (9.3%)	3 (6.0%)
	trauma	S05.8, S05.2, S05.3, T26.6	4 (4.3%)	2 (4.7%)	2 (4.0%)
	uveitis	H15.0, H22.1	2 (2.2%)	2 (4.7%)	0
	other	T85.3	1 (1.1%)	0	1 (2.0%)

*AION* anterior ischemic optic neuropathy, *AMD* age-related macular degeneration, *IOP* intraocular pressure, *NO* neuro-ophthalmology, *RAO* retinal artery occlusion, *RVO* retinal vein occlusion

Patients were asked for relevant preexisting medical conditions. Three out of four patients (72 of 93, 77.4%) indicated at least one comorbidity. Systemic arterial hypertension was the most common comorbidity and was reported in more than every second patient. Only a minority of patients had pulmonary disease, while every fifth patient was under treatment for a disorder of the heart (see online resource 2, medical history and comorbidity).

### From symptoms to ophthalmological assessment

The majority of patients (85 of 93, 91.4%) stated that from their estimate the corona pandemic did not lead to a delay in regard to their way to the tertiary center. Only eight patients acknowledged that the pandemic caused a delay to some extent. There was no statistically significant difference between the two subgroups during and after lockdown (before April 20th and from April 20th, *p* = 0.279).

Five patients indicated a delay between their decision to visit their ophthalmologist (2 vs 3 in the subgroups, *p* = 1.000), and only in 2 cases patients had to wait for more than 7 days for an appointment. After referral the study center, 90 of 93 patients were admitted within 2 days, one within 7 days and two after 8 days or more.

Most patients were referred to the tertiary center by their ophthalmologist (75 of 93, 80.6%), though the rate was significantly lower in the group after lockdown (39 of 43 vs. 36 of 50, *p* = 0.034). Referrals by the general physician (1 vs. 3, *p* = 0.623) or the telephone hotline of the Regional Association of Statutory Health Insurance Physicians (Kassenärztliche Vereinigung, 2 vs. 3, *p* = 1.00) were rare and distributed equally between groups. The fraction presenting without any contact to a referral doctor was significantly higher after April 20th (1 vs. 8, *p* = 0.036).

The majority of patients explained that their symptoms were stable or deteriorated in their waiting time for an appointment (93.5%, 87 of 93). The rate of patients with deteriorating symptoms was higher in the group after April 20th (39.5% vs 60%, 17 of 43 vs 30 of 50, *p* = 0.049). Five patients experienced problems on their way to the tertiary center. Four patients in the group during lockdown indicated problems with getting an appointment at the center (1), getting an appointment with the referral ophthalmologist (2), and “other reasons” [1]. One patient after lockdown indicated “other reasons”.

### Subjective evaluation of barriers during SARS-CoV2 pandemic

Almost 75% patients expressed that they were not or not at all afraid of a SARS-CoV2 infection. Twenty-four of the included 93 patients, however, were at least partially concerned. Similarly, 28 patients were at least partially concerned that they would be at a higher risk for an infection during their treatment at the study center. There was a statistically significant positive correlation between fear of infection and the presumption of a higher likelihood of infection at the study center (*r* = 0.445, *p* < 0.001). Fourteen patients (15.1%) were partially or rather concerned of difficulties in arranging appointments for follow-up care with their referral eye doctor.

**Fig. 1:**
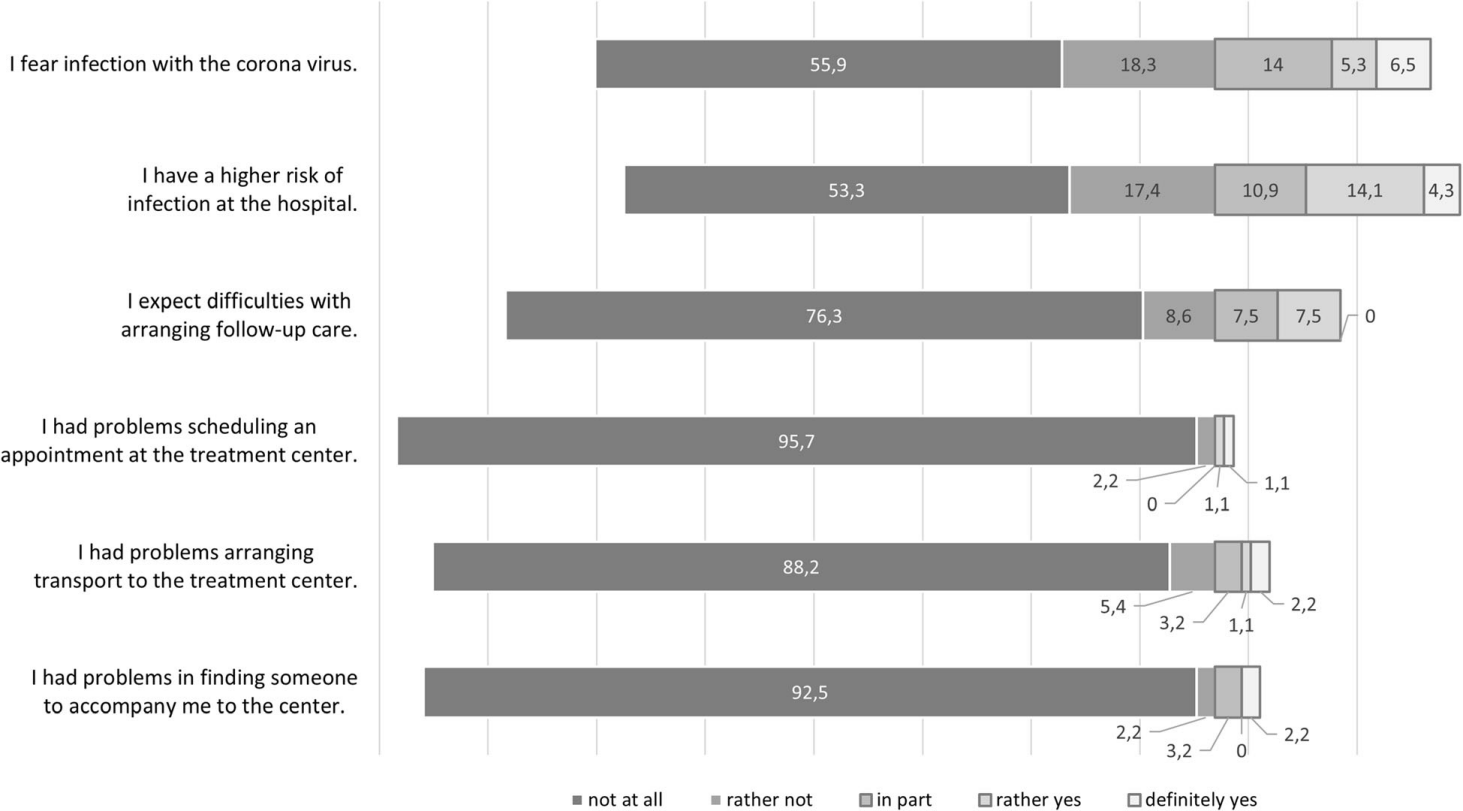
Subjective evaluation of barriers during SARS-CoV2 pandemic

Only a minority of patients indicated problems in arranging an appointment at the tertiary center (2 of 93 patients), in arranging transport to the treatment center (5 patients) or finding someone to accompany them for the examination (5 patients). The distribution of answers is shown in Fig. 1.

There was no statistically significant difference between the two subgroups during and after lockdown in any of these questions. During lockdown, patients tended to expect more problems in the follow-up care, though statistical significance was not met (Wilcoxon *p* = 0.0828).

Higher age was associated with a lower subjective fear or infection (Spearman correlation coefficient − 0.218, *p*-value 0.034), and with less concerns regarding problems with follow-up care (corr. Coefficient − 0.305, *p* = 0.003). In addition, patients of higher age were less likely to expect problems with transport (− 0.306, *p* = 0.003) or in finding someone to accompany (corr. Coefficient − 0.252, *p* = 0.015). There was no correlation of age with problems in scheduling appointments at the eye doctor (corr. Coefficient − 0.091, *p* = 0.39) or the tertiary center (corr. Coefficient − 0.14, *p* = 0.181), or the risk for an infection at the hospital (corr. Coefficient − 0.104, *p* = 0.324).

Patients with comorbidities tended to be more likely to be afraid of a COVID infection (corr. Coefficient 0.183, *p* = 0.0785) and were significantly more likely concerned about problems with organizing follow-up care (corr. Coefficient 0.222, *p* = 0.0328). There was no difference concerning fear of an infection in the hospital (0.0992, *p* = 0.347).

## Discussion

In this questionnaire study, we investigated patients’ attitude, fears and subjective barriers in regard to receiving care for urgent ophthalmologic diseases in a tertiary center in Germany.

Generally, the majority of the included patients did not report significant difficulties consulting either their referral ophthalmologist or the hospital. Only eight of the included 93 patients stated that the pandemic led to a significant delay from their decision to visit an ophthalmologist to the actual examination. During the course of the pandemic (after lockdown), however, a significantly higher ratio of patients utilized our institutions emergency unit directly without a referral by their ophthalmologist. In this group also patients had a higher likelihood of a subjective deterioration of symptoms. This might reflect difficulties in scheduling timely appointments with their referral ophthalmologist.

Other studies reported a sub-average utilization of emergency departments not only in ophthalmology [8, 9], but also in other areas like dentistry [10] or general emergency units [11]. Patients’ attitudes or subjective barriers have not been reported, though.

Retinal disorders were the most common cause for referral, with more than 60% of the cases. Within this group, retinal detachments were the most common singular cause with 41.9%, followed by retinal vascular disorders (12.9%). Comparable figures for retinal detachments have been reported from a tertiary center in the USA [12]. Corneal disorders and neuro-ophthalmologic diseases followed with 14.0 and 10.8%, respectively. While these numbers were not compared to pre-pandemic times in our study, they reflect the orientation of the tertiary center with a focus on the treatment of vitreoretinal disease.

A group from the UK found evidence for more progressed retinal detachments (characterized by a higher rate of macula-off situations and PVR) during the pandemic compared to pre pandemic levels [13]. In the presented study, patients did not report a delayed treatment, as reported above. Severity of RD was not assessed in this study. A significantly higher percentage of patients in our study group indicated that their symptoms deteriorated during the course of the pandemic, however (39.5% before vs. 60% after April 20th, *p* < 0.05).

Similar trends have been reported in other surgical areas. For example, a group from Scotland presented evidence for more progressed cases of appendicitis in a district general hospital, defined as a higher severity and the increased need for surgery [14]. A group from Italy reported that in up to 40% of non-traumatic emergency cases treatment was unusually delayed [15]. Another group from Italy reported about a delay in treatment in 12 children with acute diseases, resulting in the need of intensive care in 6 patients and death in 4 of these patients [16]. The reasons for the reported delays cannot be entirely elucidated. Contributing factors may be associated with different complexes, such as the patients’ attitudes (e.g. fear of infection), institutional guidelines (e.g. limited access to emergency units, general physicians), or governmental regulations (lockdown).

In our study, at least every fourth patient was afraid of COVID-19, and every third was concerned that they had a higher likelihood of infection at the treatment center. During the conception of the study we hypothesized, that anxiety and fear of infection would be more dominant during the early phases of the disease, possibly also due to the changes in the general recommendations for hospital admission and to general lockdown. This was reflected in the study design by subdividing the study group in to two subgroups (during and after lockdown, which ended April 20th, 2020). However, there was no difference in regard to the two subgroups in terms of anxiety. Patients with systemic comorbidity or preexisting health conditions tended to be more afraid of an infection and were more likely to expect problems in the follow-up care. Compared to a study investigating a representative sample of US adults, which reported an average of nearly 7 in a scale of 10 for fear [17], the patients in our study appeared less concerned. This may correlate with the time of survey, as well as with the extent of the pandemic in Germany and the USA. Figures were generally lower in Germany compared to the US during the first wave of the pandemic. Additionally, the urgent medical condition might also change the perception of personal risk, compared to a randomly selected study group.

In this study, higher age was negatively correlated with fear of infection itself and the risk for infection in the hospital. Similar findings were reported in an online survey of residents of Hong Kong [18], where younger age was statistically significantly correlated with a higher concern of becoming infected in a multivariate analysis as the only significant factor. This study, however, did not find an association of a lower perceived personal risk with less careful behavior (e.g. hand-washing, avoidance of public gatherings) in the older patients. A national survey in the US done in March 2020 also reported a generally more optimistic outlook and better mental health in older patients, except the perceived infection-fatality risk [19]. Still, protection of high-risk groups, such as patients aged 65 or older, should be protected, and the health care providers should establish protocols minimizing contacts of patients at risk, either with health care providers or other patients [3, 20, 21]. Prioritizing of the elderly for vaccinations will be an additional important means in reducing risks in this group.

The presented study has some limitations which have to be considered. Firstly, this is a study from a singular tertiary center in northern Germany. Northern Germany was less affected during the first wave of COVID-19, and thus limitations in access to the health system may differ significantly across different regions. While the results of this study may help to gather an impression of aspects important for patients, they cannot be generalized and have to be carefully interpreted.

We included patients with an acute need for treatment as an inpatient, regardless of whether surgery was indicated or not. This has to be considered when comparing the numbers from this setting to other studies reporting on emergency department visits [8, 9] or ophthalmic surgical care [12]. Institutional guidelines changed during the first phase of the pandemic, reflecting the uncertainties of risk for infection, the need for personnel and capacity for COVID patients at the university hospital, and the time needed to establish appropriate protocols. Elective examinations and surgery were suspended during general lockdown (before April 20th) and allowed again thereafter. We reflected these changes in policy by dividing the study group into two subgroups around April 20th (during and after lockdown).

Patient recruitment was in part performed retrospectively, and due to the design of the study especially patients in the pre April 20th subgroup were interviewed via telephone. This may result in a recall bias and may limit the validity of the analysis [22]. However, the retrospective group was included despite the risk for bias, as especially in the first phase of the pandemic there was a high level of uncertainty in the general public and among physicians, possibly leading to delayed treatment. Some patients eligible for the study did not sign informed consent and could not be included. Of note, institutional or national guidelines in which patients should be treated as inpatients may differ and could hinder comparability of the provided numbers with other countries or health systems. Patients who did not visit their ophthalmologist or referred to our emergency unit at all could not be included.

## Conclusion

This study analyzed the patient’s perspective on barriers on their way to treatment of urgent ophthalmic diseases during the early phase of the SARS-CoV-2 pandemic in Germany. Generally, most patients encountered no problems on their way to our tertiary ophthalmology center. However, about one third of patients was afraid of a COVID-19 infection, and saw a potentially higher risk for infection at the center. Fear of infection was significantly less dominant in older patients, although mortality is higher in this group. Efforts have to be made to reduce risk of infection in higher risk groups.

## Supplementary Information

**Additional file 1: SOM 1.** Questionnaire (german)

**Additional file 2: SOM 2.** Questionnaire (english)

**Additional file 3: SOM 3.** Table medical history and comorbidity

## Data Availability

The datasets generated and/or analyzed during the current study are not publicly available due to data protection guidelines, but can be provided by the corresponding author if needed.

## Abbreviations

AION: Anterior ischemic optic neuropathy
AMD: Age-related macular degeneration
COVID-19: Corona virus disease, 2019
IOP: Intraocular pressure
NO: Neuro-ophthalmologic
PVR: Proliferative vitreoretinopathy
RAO: Retinal arterial occlusion
RRD: Rhegmatogenous retinal detachment
RVO: Retinal venous occlusion
SARS-CoV-2: Severe acute respiratory syndrome, coronavirus 2
Corr. Coefficient: Spearman correlation coefficient
