# Supplementary material for: Patients’ perspective on emergency treatment of ophthalmologic diseases during the first phase of SARS-CoV2 pandemic in a tertiary referral center in Germany – the COVID-DETOUR questionnaire study
Source: BMC Ophthalmol. 2021 Aug 16;21:301. doi: 10.1186/s12886-021-02054-7 (PMC8366159; doi:10.1186/s12886-021-02054-7)
Supplement: Supplementary file 1 — Additional file 1: SOM 1. Questionnaire (german) [file 12886_2021_2054_MOESM1_ESM.pdf]

Fragebogen zur Erfassung einer Verzögerung der Augenärztlichen Notfallversorgung während der SARS-CoV-2-Pandemie

(COVID-19 DETOUR: Delayed Treatment of urgent ophthalmologic treatment during SARS-CoV-2 pandemic)

Identifikationsnummer: _________

Bitte beantworten Sie folgende Fragen nach bestem Wissen und Gewissen.

1. Haben Sie aufgrund der aktuellen SARS-CoV-2-Pandemie einen Arztbesuch herausgezögert?

   Kreuzen Sie den Wert an, der am ehesten zutrifft.

Trifft nicht zu (1)	Trifft eher nicht zu (2)	teils – teils (3)	Trifft eher zu (4)	Trifft zu (5)
☐	☐	☐	☐	☐

2. Auf wessen Empfehlung haben Sie sich in der Klinik für Augenheilkunde vorgestellt?

   ☐ Überweisung durch den Augenarzt.

   Wenn ja, um welchen Augenarzt handelte es sich?

   ☐ Eigener Augenarzt.

   ☐ Vertretung.

   ☐ Überweisung durch den Hausarzt.

   ☐ Empfehlung durch die Telefonhotline der Kassenärztlichen Vereinigung.

   ☐ Empfehlung von Angehörigen, Freunden o.ä..

   ☐ Direkt, ohne Empfehlung anderer.

3. Warum haben Sie sich dann schließlich zum Arztbesuch entschieden?

   ☐ Die Beschwerden sind verschwunden, ich wollte aber Sicherheit.

   ☐ Die Beschwerden sind besser geworden, aber nicht verschwunden.

   ☐ Es gab keine Verbesserung der Beschwerden.

   ☐ Die Beschwerden haben zugenommen.

4. Gab es Verzögerungen zwischen Ihrer Entscheidung, zum Arzt zu gehen, und der augenärztlichen Untersuchung?

   ☐ Nein.

   ☐ Ja, und zwar…(Mehrfachantworten sind möglich)

      ☐ … bis zur Untersuchung beim Augenarzt.

      ☐ … bis zur Untersuchung in der Augenklinik.

      ☐ … durch mangelnde Transportmöglichkeiten.

      ☐ … durch fehlende Unterstützung (Angehörige, Freunde…)

      ☐ … aus anderen Gründen.

5. Wie lange dauerte es, bis Sie einen Termin beim Augenarzt bekamen?

   ☐ Sofort (innerhalb von 1-2 Tagen).

   ☐ Innerhalb von ≤7 Tagen.

   ☐ Innerhalb von 8-14 Tagen.

   ☐ Länger als 2 Wochen.

   ☐ Nicht zutreffend.

6. Wie lange dauerte es, bis Sie in der Klinik für Augenheilkunde untersucht wurden?

   ☐ Sofort (innerhalb von 1-2 Tagen).

   ☐ Innerhalb von ≤7 Tagen.

   ☐ Innerhalb von 8-14 Tagen.

   ☐ Länger als 2 Wochen.

7. Welche Aussagen treffen für Sie zu?

   Kreuzen sie jeweils den am besten passenden Wert an.

a) *Ich habe Sorge, mich mit dem Corona-Virus zu infizieren.*

Trifft nicht zu (1)	Trifft eher nicht zu (2)	teils – teils (3)	Trifft eher zu (4)	Trifft zu (5)
☐	☐	☐	☐	☐

b) *Ich habe ein erhöhtes Risiko, mich in der Klinik zu infizieren.*

Trifft nicht zu (1)	Trifft eher nicht zu (2)	teils – teils (3)	Trifft eher zu (4)	Trifft zu (5)
☐	☐	☐	☐	☐

c) *Es war schwierig, einen Termin beim Augenarzt zu bekommen.*

Trifft nicht zu (1)	Trifft eher nicht zu (2)	teils – teils (3)	Trifft eher zu (4)	Trifft zu (5)
☐	☐	☐	☐	☐

d) *Es war schwierig, einen Termin in der Klinik zu bekommen.*

Trifft nicht zu (1)	Trifft eher nicht zu (2)	teils – teils (3)	Trifft eher zu (4)	Trifft zu (5)
☐	☐	☐	☐	☐

e) *Ich hatte Schwierigkeiten, den Transport in die Klinik zu organisieren.*

Trifft nicht zu (1)	Trifft eher nicht zu (2)	teils – teils (3)	Trifft eher zu (4)	Trifft zu (5)
☐	☐	☐	☐	☐

f) *Ich hatte Schwierigkeiten, eine Begleitperson zu organisieren.*

Trifft nicht zu (1)	Trifft eher nicht zu (2)	teils – teils (3)	Trifft eher zu (4)	Trifft zu (5)
☐	☐	☐	☐	☐

g) *Ich mache mir Sorgen, dass die Nachbetreuung durch die SARS-CoV-2 Pandemie schwieriger wird.*

Trifft nicht zu (1)	Trifft eher nicht zu (2)	teils – teils (3)	Trifft eher zu (4)	Trifft zu (5)
☐	☐	☐	☐	☐

Fragebogen zur Erfassung einer Verzögerung der Augenärztlichen Notfallversorgung während der SARS-CoV-2-Pandemie

Allgemeine Angaben

Diagnose: ______________________

Beginn Symptome: ______________________ (TT/MM/JJJJ)

Termin Augenarzt: ______________________ (TT/MM/JJJJ)

Vorstellung Augenklinik: ______________________ (TT/MM/JJJJ)

Datum Fragebogen: ______________________ (TT/MM/JJJJ)

Alter des Patienten: ______________________ Jahre

Geschlecht des Patienten: □ weiblich □ männlich

Eingeschränkte Mobilität: □ ja □ nein

Komorbidität:

□ Diabetes mellitus

□ arterielle Hypertonie

□ pulmonale Erkrankung

□ Herzerkrankung

□ Nikotin (wenn ja: ________ pack years)

□ andere
