# Supplementary material for: Patients’ perspective on emergency treatment of ophthalmologic diseases during the first phase of SARS-CoV2 pandemic in a tertiary referral center in Germany – the COVID-DETOUR questionnaire study
Source: BMC Ophthalmol. 2021 Aug 16;21:301. doi: 10.1186/s12886-021-02054-7 (PMC8366159; doi:10.1186/s12886-021-02054-7)
Supplement: Supplementary file 2 — Additional file 2: SOM 2. Questionnaire (english) [file 12886_2021_2054_MOESM2_ESM.pdf]

Questionnaire to detect a delay in the treatment of urgent ophthalmologic diseases during the SARS-CoV-2-pandemic

(COVID-19 DETOUR: Delayed Treatment of urgent ophthalmologic treatment during SARS-CoV-2 pandemic)

Identification numer: _________

Please answer these questions to your best knowledge.

1. Did you delay your appointment at your ophthalmologist due tot he COVID-19 pandemic?

   Please check the answer best suitable.

Not at all (1)	Rather not (2)	In part (3)	Mostly agree (4)	Totally agree(5)
☐	☐	☐	☐	☐

2. Who advised you to go to the hospital?

   ☐ Referral by ophthalmologist.

   If yes: which ophthalmologist?

   ☐ Own ophthalmologist.

   ☐ Different ophthalmologist,

   ☐ Referral by general physician.

   ☐ Recommendation by the telephone hotline of the Regional Association of Statutory Health Insurance Physicians.

   ☐ Recommendation by relatives, friends, etc

   ☐ Without recommendation.

3. Why did you decide to visit your ophthalmologist?

   ☐ The symptoms are gone, but I wanted to be sure.

   ☐ The symptoms improved but did not resolve.

   ☐ The symptoms did not improve.

   ☐ The symptoms deteriorated.

4. Was there a delay between your decision to have your eye examined and the actual examination?

   ☐ No.

   ☐ Yes, … (multiple answers are allowed)

      ☐ … until I was examined by my ophthalmologist.

      ☐ … until I had an appointment at the hospital.

      ☐ … due to problems with a transfer tot he hospital.

      ☐ … due to lack of support by friends, family, etc.

      ☐ … for other reasons.

5. How soon could an appointment at your ophthalmologist be arranged?

   ☐ Immediately (within 2 days).

   ☐ Within ≤7 days.

   ☐ Within 8-14 days.

   ☐ More than 2 weeks.

   ☐ Not applicable.

6. How soon could an appointment at the hospital be arranged?

   ☐ Immediately (within 2 days).

   ☐ Within ≤7 days.

   ☐ Within 8-14 days.

   ☐ More than 2 weeks.

7. Which statements apply?

   Please check the best suitable answer.

a) *I am afraid of getting infected with the coronavirus.*

Not at all (1)	Rather not (2)	In part (3)	Mostly agree (4)	Totally agree(5)
☐	☐	☐	☐	☐

b) *I have a greater risk of infection during my stay at the hospital.*

Not at all (1)	Rather not (2)	In part (3)	Mostly agree (4)	Totally agree(5)
☐	☐	☐	☐	☐

c) *I had difficulties arranging an appointment at the ophthalmologist.*

Not at all (1)	Rather not (2)	In part (3)	Mostly agree (4)	Totally agree(5)
☐	☐	☐	☐	☐

d) *I had difficulties arranging an appointment at the hospital.*

Not at all (1)	Rather not (2)	In part (3)	Mostly agree (4)	Totally agree(5)
☐	☐	☐	☐	☐

e) *I had difficulties arranging transport tot he hospital.*

Not at all (1)	Rather not (2)	In part (3)	Mostly agree (4)	Totally agree(5)
☐	☐	☐	☐	☐

f) *I had difficulties organizing someone to accompagny me tot he hospital.*

Not at all (1)	Rather not (2)	In part (3)	Mostly agree (4)	Totally agree(5)
☐	☐	☐	☐	☐

g) *I am afraid that arranging follow-up care will be difficult due to the pandemic.*

Not at all (1)	Rather not (2)	In part (3)	Mostly agree (4)	Totally agree(5)
☐	☐	☐	☐	☐

Questionnaire to detect a delay in the treatment of urgent ophthalmologic diseases during the SARS-CoV-2-pandemic

General information

diagnosis: ________________________

onset of symptoms: ________________________ (TT/MM/JJJJ)

appointment ophthalmologist:________________________ (TT/MM/JJJJ)

appointment hospital: ________________________ (TT/MM/JJJJ)

date of questionnaire: ________________________ (TT/MM/JJJJ)

patient age: ________________________ years

patient sex: □ female □ male

impaired mobility: □ yes □ no

systemic comorbidity:

□ diabetes mellitus

□ systemic arterial hypertension

□ pulmonary disease

□ heart disease

□ smoking (if yes: _________ pack years)

□ other
