# Supplementary material for: Patients’ perspective on emergency treatment of ophthalmologic diseases during the first phase of SARS-CoV2 pandemic in a tertiary referral center in Germany – the COVID-DETOUR questionnaire study
Source: BMC Ophthalmol. 2021 Aug 16;21:301. doi: 10.1186/s12886-021-02054-7 (PMC8366159; doi:10.1186/s12886-021-02054-7)
Supplement: Supplementary file 3 — Additional file 3: SOM 3. Table medical history and comorbidity [file 12886_2021_2054_MOESM3_ESM.pdf]

	total study group	during lockdown<br><br>(before April 20th)	after lockdown<br><br>(from April 20th)
diabetes	14 (5.1%)	5 (11.6%)	9 (18.0%)
systemic arterial hypertension	49 (52.7%)	19 (44.2%)	30 (60.0%)
pulmonary disease	7 (7.5%)	4 (9.3%)	3 (6.0%)
heart disease	19 (20.4%)	7 (16.3%)	12 (24.0%)
smoking	23 (24.7%)	10 (23.3%)	13 (26.0%)
other	32 (34.4%)	19 (44.2%)	13 (26.0%)
at least 1 comorbidity	72 (77.4%)	35 (81.4%)	37 (74.0%)
reduced mobility	17 (18.5%)	8 (18.6%)	9 (18.0%)

Table: medical history and comorbidity
